# YES, a novel therapeutic target in hepatocellular carcinoma

**DOI:** 10.1080/23723556.2022.2069993

**Published:** 2022-05-01

**Authors:** Marjorie Lapouge, Sylvain Meloche

**Affiliations:** a Signaling and Cell Growth Laboratory, Institute for Research in Immunology and Cancer, Montreal, Quebec, Canada; b Department of Pharmacology and Physiology, Université de Montréal, Montreal, Quebec, Canada; c Molecular Biology Program, Faculty of Medicine, Université de Montréal, Montreal, Quebec, Canada

**Keywords:** YES, SRC-family kinases, YAP/TAZ, hepatocellular carcinoma, kinase inhibitors

## Abstract

Identification of dominant, actionable oncogenic signaling pathways is key to guide the development of new targeted treatments for advanced-stage hepatocellular carcinoma (HCC). We have recently unveiled a novel YES-YAP/TAZ signaling axis involved in liver cancer development. Our study identifies the tyrosine kinase YES as a potential therapeutic target in HCC.

Liver cancer is the sixth most common cancer and the third leading cause of cancer-related deaths worldwide, with hepatocellular carcinoma (HCC) accounting for ~90% of cases.^1^ Contrary to other cancers, the incidence of HCC is increasing in developed countries as a result of the high prevalence of hepatitis C virus-induced cirrhosis and the rise of nonalcoholic fatty liver disease (NAFLD). HCC is a highly heterogeneous malignancy with a complex pathophysiology.^1,2^ Integrative genomic analyses have defined the landscape of genetic alterations in HCC, enabling the classification of the disease into two molecular subtypes.^1,3^ The proliferation class, which is characterized by activation of oncogenic signaling pathways, *TP53* mutations, and expression of progenitor cell markers, is associated with a more aggressive phenotype. The nonproliferation class, which retains features of normal hepatocytes and is enriched for alterations in the Wnt–β-catenin pathway, has a better outcome. However, this molecular knowledge has not influenced clinical practice. Only approximately 25% of HCCs display potentially actionable mutations, and no mutation is used in clinical-decision making. Unfortunately, a majority of HCC patients are still diagnosed at a late stage, making them ineligible for curative surgical interventions. The multi-kinase inhibitors sorafenib and lenvatinib, and the combination of atezolizumab/bevacizumab are the only first-line therapies approved for advanced HCC, with a modest gain in overall survival.^1^ There is therefore an urgent need to identify novel targeted therapies for advanced-stage HCC patients.

As an approach to identify novel protein kinases controlling liver cancer cell proliferation, we performed an unbiased loss-of-function shRNA screen of the human kinome in HCC cells and identified the SRC-family kinase (SFK) YES as the top-scoring hit.^4^ SFKs are a family of non-receptor tyrosine kinases that play a key role in transducing signals from multiple receptors to regulate cell proliferation, differentiation, survival, cytoskeleton dynamics, and motility.^5^ The activity of SFKs is deregulated in many types of cancers, and this has been associated with the development, progression, and metastatic dissemination of the tumors.^6,7^ YES is a ubiquitously expressed member of the SFKs whose specific role in cancer remains poorly understood. The activity of YES has not been linked to HCC pathogenesis. We therefore rigorously validated the essential role of YES in HCC proliferation using a combination of genetic and pharmacological perturbations. We also showed that the function of YES is generalizable to multiple human HCC cell lines.

To address the role of YES in HCC development *in vivo*, we injected wild type and *Yes1-/-* mice with the hepatocarcinogen diethylnitrosamine, which induces liver tumors with a high penetrance. We found that genetic disruption of YES drastically reduced the incidence, number and size of liver tumor nodules in this model. Notably, inactivation of YES did not interfere with normal hepatocyte proliferation and liver regeneration, suggesting that YES activity is required specifically for the proliferation of transformed hepatocytes. To determine if YES activation is sufficient to induce HCC formation, we expressed the constitutively-activated mutant YES Y537F in mouse hepatocytes by hydrodynamic gene delivery. We observed that transgenic expression of active YES is sufficient to induce HCC formation in mice with full penetrance. These findings identify YES as an oncogenic driver in liver cancer.^4^ To evaluate the cell-autonomous role of YES in sustaining the proliferation of HCC cells *in vivo*, we engineered HCC cells with an inducible *YES1* shRNA and transplanted the cells in immunodeficient mice. Hepatocyte-specific depletion of YES completely abolished HCC growth, associated with a reduction in cell proliferation and increased apoptosis. Treatment with the SFK inhibitor dasatinib also induced tumor regression in the diethylnitrosamine HCC model. Thus, YES activity is both necessary and sufficient for HCC formation in mice.^4^

Elucidation of the signaling network of YES is critical to understand its oncogenic role and to identify molecular biomarkers predictive of YES dependency in cancer. By analyzing the transcriptomes of a series of HCC cell lines subjected to selective pharmacological inhibition of YES kinase activity, we found that the list of differentially expressed genes was enriched for targets of the transcriptional co-activators YAP (YAP1, Yes-associated protein 1, best known as YAP) and TAZ (WWTR1, WW domain-containing transcription regulator protein 1, best known as TAZ).^8^ The importance of YAP/TAZ in cancer, including liver cancer, is well established^,9^which prompted us to investigate the connection of YES signaling to YAP/TAZ. We showed that YES directly phosphorylates YAP and TAZ, promoting their nuclear accumulation and transcriptional activity in HCC cells (Figure 1). SRC was also shown to phosphorylate YAP and regulate its nuclear distribution in keratinocytes^,10^ suggesting possible functional redundancy of different SFKs. Genetic and pharmacological manipulation of YES activity also modulated the nuclear localization of YAP and the expression of YAP/TAZ target genes in liver tumors, demonstrating that this mechanism operates *in vivo*. Importantly, we showed that YAP/TAZ are effectors of YES-induced hepatocyte transformation in mice.^4^

**Figure 1.**
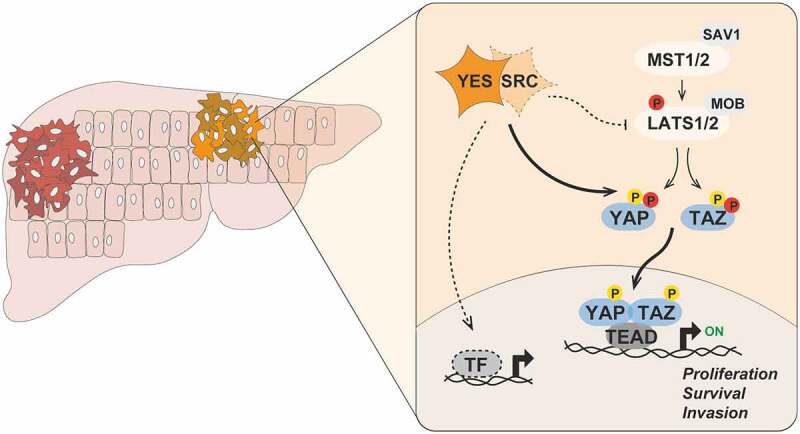
Aberrant YES signaling promotes hepatic oncogenesis. Upon activation in hepatocytes, the SRC-family kinase YES directly phosphorylates YAP (Tyr391 and Tyr407) and TAZ (Tyr305) on tyrosine residues, thereby promoting their nuclear accumulation and transcriptional activity. SRC may also redundantly regulate the activity of YAP and TAZ in certain cellular contexts. In parallel, YES and SRC can phosphorylate and inhibit the activity of LATS1/2 kinases to antagonize the negative regulation of YAP/TAZ by the Hippo pathway. Activated YAP and TAZ then associate with TEAD, and possibly other, family of transcription factors to activate gene expression programs controlling proliferation, survival and invasion of transformed hepatocytes. It is likely that YES signaling regulates the activity of other transcription factors and effectors that contribute to liver tumorigenesis. Phosphorylated tyrosine and serine/threonine residues are depicted in yellow and red, respectively. MST, mammalian STE20-like protein kinase; SAV1, Salvador family WW domain containing protein 1; LATS, large tumor suppressor kinase; MOB, MOB kinase activator; YAP, Yes-associated protein 1; TAZ, WW domain-containing transcription regulator protein 1; TEAD, TEA domain; TF, transcription factor.

Analysis of human HCC samples revealed that SFKs are hyperactivated in ~25% of primary HCC cases, correlating with the tyrosine phosphorylation and nuclear accumulation of YAP. To address the specific role of YES, we derived a gene signature of active YES from our transcriptomic datasets of HCC cell lines. We showed that high YES activity, rather than *YES1* mutation or increased YES abundance, predicts shorter overall survival in HCC patients, underscoring the translational relevance of our findings.^4^

In summary, we have identified a novel YES oncogenic signaling pathway in HCC that has escaped previous large-scale genomic and proteomic studies. The observation that this pathway is activated in a subset of human HCC provides a strong rationale for targeting YES in liver cancer. Notably, the identification of YAP/TAZ as downstream effectors of YES oncogenesis offers new opportunities for the validation of predictive biomarkers of efficacy to future YES-targeted therapies. The high frequency of YAP/TAZ deregulation in multiple human cancers^9^ also suggest that pharmacological inhibition of YES may find clinical usefulness in other solid cancers.
